# Proceedings of the Ohio State Dental Association

**Published:** 1868-02

**Authors:** G. W. Keely


### PROCEEDINGS OF THE OHIO STATE DENTAL ASSOCIATION.

SOCIETY met pursuant to adjournment at the rooms of the Young Men's Christian Association, Columbus, December 24th. A quorum not being present, no regular morning session was held.

AFTERNOON SESSION.

President Dr. George Watt called the meeting to order.

The Secretary being absent, Dr. G. W. Keely was appointed *pro tem.*

Minutes of the last annual meeting read and approved.

Roll of members being called, the following answered to their names: G. W. Keely, R. Corson, B. T. Spellman, J. W. Wortman, J. B. Beauman, George Watt, A. A. Blount, N. W. Williams, D. R. Jennings, J. Taft, G. W. Dunn, J. Fowler, C. H. Harroun.

Drs. Harroun and Jennings were appointed to fill the vacancy in the Committee on Membership.

The following was offered: "At the next meeting I will propose that the By-laws be so amended as to provide that the annual meeting shall be held on the first Tuesday of December each year; and that the semi-annual meetings be discontinued." Signed A. A. BLOUNT.

A letter from Dr. A. Berry was read, regretting his inability to be present, which, on motion, was received and ordered placed on file.

Drs. Blount, Dunn and Corson were appointed to nominate candidates for officers for the ensuing year.

Report received and laid on the table.

Drs. Williams and Corson were appointed to audit the Treasurer's account, and reported the same correct, with balance of $50.12 in the Treasury.

Committee on Membership recommended Drs. John H. Paine, of Middletown, and George L. Paine, of Xenia, for active members, and Dr. S. C. Taylor, of Toledo, as an honorary member, all of whom were duly elected.

Report of Nominating Committee taken up, and the following officers elected:

President — J. Taft.

Vice Presidents — C. H. Harroun, 1st; C. R. Butler, 2d.

Corresponding Secretary — N. W. Williams.

Recording Secretary — A. W. Maxwell.

Treasurer — A. Berry.

Dr. J. Taft, President elect, took the chair, making a few appropriate remarks. On motion of Dr. Watt, members elected at the last semi-annual meeting required to pay but one-half the yearly dues.

On motion, the By-laws fixing the time for the annual meeting were suspended by unanimous vote, after which it was resolved to hold the next annual meeting, commencing at 10 o'clock A. M., on the first Tuesday in December.

The following resolution was submitted by Dr. Spellman and passed:

*Resolved*, That any members now in debt to this society, who shall continue so until the next meeting, shall appear in person or by proxy at the same, and show cause for delinquency, and those failing to do so shall cease to be members; and that the Treasurer be requested to notify delinquents.

The rubber question was taken up and discussed fully by Dr. Taft, and spoken upon by Drs. Keely, Spellman, Harroun, and others, and a determination shown to contest the matter to the last end.

Society then adjourned to meet at 7 o'clock.

G. W. KEELY, *Secretary, pro tem.*

EVENING SESSION.

Society met, President Taft in the chair. Minutes of afternoon meeting read and approved.

On motion, Drs. Watt and J. H. Paine were appointed to select delegates to the American Dental Association. The following members were proposed and duly appointed: Drs. B. A. Rose, H. M. Edson, C. H. Harroun, R. Corson, John Fowler, H. C. Howells, G. W. Wortman, W. R. Lilly, A. W. Maxwell, T. W. French, A. Terry, L. Buffett.

The Bill to regulate the practice of Dentistry in the State, was taken up and discussed by Taft, Spellman, Keely, Paine, Watt, Harroun, and others. When, on motion, Drs, Harroun, Watt, and Jennings were appointed to select a committee of fifteen, the duty of which shall be to look after the interest of the Bill at the coming session of the General Assembly. The following were chosen; J. Taft, W. P. Horton, J. B. Beauman, W. H. Herriott, G. L. Paine, F. S. Whitslar, F. H. Rehwinkle, J. M. Rhoades, G. W. Keely, M. DeCamp, A. W. Maxwell, J. A. Paine, H. R. Smith, A. A. Blount, C. H. Harroun.

The Executive Committee reported the following subjects for discussion: First, necrosis of alveolar processes and treatment; second, preparation of cavities for filling; third, means for controlling flow of saliva; fourth, Dental medicines and anæsthetics; fifth, mechanical Dentistry.

The first subject was then taken up and discussed at considerable length by Harroun, Keely, Taft, Blount, and a few others. It being late and the design of the society to adjourn this evening, the other subjects were laid over.

The President announced the standing committees for the ensuing year to be as follows: M. DeCamp, J. W. Wortman, J. Fowler, Executive Committee.

B. T. Spellman, C. H. Harroun, J. H. Paine, Membership.

N. W. Williams, F. H. Rehwinkle, G. W. Keely, Dental Ethics.

George Watt, G. L. Paine, A. A. Blount, Publication.

The President appointed the following gentlemen, essayists for the next meeting: Drs. H. A. Smith, A. Berry, C. R. Butler, J. H. Paine, and C. H. Harroun.

The following was adopted:

*Resolved*, That the automatic plugger, submitted for examination by Dr. S. C. Taylor, of Toledo, is, in our opinion, superior to any one heretofore exhibited to this society.

J. Taft's bill of $22 50 for printing, and a bill for $5 for use of hall was allowed, and ordered paid.

Although but few members were present, all felt their time had been profitably spent, and that it was "good to be here."

After a short season of social intercourse the society adjourned. G. W. KEELY, *Secretary, pro tem.*

